# Antiseptic Osteotomy of the Tibia; Rapidly Fatal Carbolic Intoxication

**Published:** 1881-09

**Authors:** 


### Antiseptic Osteotomy of the Tibia; Rapidly Fatal Carbolic Intoxication.

Mr. Pearce Gould, at a recent meeting of the Clinical Society of London (*Lancet*, May 21, 1881), read a paper on a Case of Antiseptic Osteotomy of the Tibia, in which rapidly fatal carbolic intoxication occurred. A lad, eight years of age, of robust appearance, was admitted, under his care, into Westminster Hospital in Oct., 1880, with rickety deformity of the legs, especially of the left tibia. Other treatment had been perseveringly tried at the National Orthopædic Hospital for some time without avail, and he was sent in for the purpose of having osteotomy performed. His general health was very good. On Oct. 30th chloroform was administered, and the left tibia divided with McEwen's chisel, with a careful observance of the details of Listerism. He quickly recovered from the effects of the chloroform, and slept well through the night; next morning (Oct. 31st) he took his breakfast and appeared quite well; temperature 98.4°. At 11 A. M. (twenty-four hours after the operation) he vomited; and vomited a watery fluid repeatedly up till 9.30 P. M. He also passed two orange-colored loose stools. At 7 P. M. he was rigid, and at 8 P. M. very restless; at 9 P. M. he was almost unconscious; he was breathing hurriedly; pulse almost imperceptible at the wrist. At 10 P. M. Mr. Gould saw him; he was then conscious; lying quiet and quite flat; respiration 44 per minute; air entered lungs freely; pulse very small and rapid, pupils small. He died, asphyxiated, at 2.30 A. M., Nov. 1st, thirty-six hours and three-quarters after the operation. There was complete suppression of urine after 11 A. M. Temperature rose to 100.2°, and fell to 99.6° at 2 A. M. on Nov. 1st. The treatment consisted in the application of warmth and stimulants to the surface, and brandy internally. At the autopsy all the viscera were apparently healthy, with the exception of congestion of the tracheal and bronchial mucous membrane, and excess of mucous in these tubes, and slight congestion of the lungs. The left side of the heart was empty, the right side contained partly-clotted blood; blood in smaller veins all fluid, in the larger partly in soft clots, all of it black. No petechiæ; no staining of endocardium or intima; no sign of fat embolism; bladder empty. A small bloodclot in the wound; no crushing of the medulla; no thrombosis of tibial vessels. Microscopic preparations of the organs were made for Mr. Gould by Dr. Heneage Gibbes, some of which were shown at the meeting. From these it was seen that there was thrombosis of the capillaries and smaller vessels of the lungs with minute spots of round-cell inflammatory exudation. Similar minute inflammatory foci were also found in the kidneys, while sections of the stomach showed intense inflammation of its mucous surface, only traces of its glands being visible, and at places the process was evidently advancing to suppuration. No sign of fat embolism.

In discussing the cause of death Mr. Gould pointed out that shock, chloroform, the acute specifics, erysipelas, septicæmia, pyæmia, and fat embolism might be excluded, as was also the ingestion of any irritant matter, but yet both the symptoms and pathological changes showed evidence of the circulation of some irritant in the blood. The symptoms were individually considered, and it was shown that the vomiting, diarrhœa with orange stools, collapse, rigidity, restlessness, loss of consciousness, very hurried respiration, slight rise of temperature, and death from asphyxia, were the symptoms observed in cases of undoubted carbolic intoxication. The urine passed before the onset of vomiting appeared to be normal, and was not preserved; the suppression of urine later on was a new fact in such cases. The softly coagulated and black blood had been noted before, but there was no record of any microscopical examination of the apparently healthy organs usually found. The quantity of acid absorbed was no doubt small, and the great susceptibility of the patient to its toxic effect must at present be spoken of as an idiosyncrasy; but from the absence of other cause, and of any facts to negative the supposition, as well as the agreement of the symptoms and post-mortem pathological changes in the main with those observed in cases of carbolic intoxication, he was forced to the conclusion that such poisoning was the cause of death. Prof. Lister remarked upon the interest attaching to the suppression of urine, which he had not met with before. He had no doubt that Mr. Gould's view must be accepted, that this patient was one of those who had an unfortunate susceptibility to the toxic effects of carbolic acid. He had met with cases of such susceptibility, and one he called to mind was a lady whose breast he removed and dressed in his usual manner; intense symptoms of carbolic poisoning came on, and boracic lint was substituted for the gauze dressing, but the spray was used when the lint was changed, and this was sufficient to keep up the symptoms, which only abated when its use was discontinued. As an instance of the special local susceptibility of some patients, he mentioned a case of chronic synovitis of the knee-joint, which he opened and dressed antiseptically, and next day he found all the skin under the gauze intensely red and irritated. It had been stated that there was no antiseptic that could with safety replace carbolic acid, though several had been tried; but he was in a position to say that the oil of Eucalyptus globulus, if used properly, is a powerful and perfectly reliable antiseptic, and is also quite unirritating and without toxic effects. If the oil be made into an emulsion with spirit and water, and then used, it very quickly evaporates; but he had found that gum damar holds it exceedingly well, and the mixture remains soft and strongly odorous of the oil even at the end of several weeks. He had used a gauze prepared with a mixture of one part of the oil, three of damar, and three of paraffine, and he was able to assert that it might be thoroughly trusted as an antiseptic where carbolic acid was unadvisable.—*Medical Abstract.*

